# Kallikrein gene family as biomarkers for recurrent prostate cancer

**DOI:** 10.3325/cmj.2020.61.450

**Published:** 2020-10

**Authors:** Fatma Busra Boyukozer, Esra Guzel Tanoglu, Mustafa Ozen, Michael Ittmann, Elif Sibel Aslan

**Affiliations:** 1 Department of Molecular and Medical Genetics, Biruni University, Istanbul, Turkey; 2 Department of Molecular Biology and Genetics, University of Health Sciences, Istanbul, Turkey; 4 Department of Pathology and Immunology, Baylor College of Medicine, Houston, TX; 5 Department of Molecular Biology and Genetics, Biruni University, Istanbul, Turkey

## Abstract

**Aim:**

To assess kallikrein (*KLK*) expression in recurrent and non-recurrent prostate tumors and adjacent healthy prostate tissues.

**Methods:**

The expression levels of 15 *KLK* genes in 34 recurrent and 36 non-recurrent prostate cancer samples and 19 adjacent healthy prostate tissue samples was assessed with quantitative reverse-transcription polymerase chain reaction. The samples were obtained from Baylor College of Medicine, Houston, TX, USA between 2013 and 2016 .

**Results:**

Compared with controls, prostate cancer samples showed a strong decrease in *KLK1, KLK4, KLK9,* and *KLK14*. Recurrent samples were negative for *KLK1*, *KLK2,* and *KLK14* but demonstrated higher levels of *KLK3, KLK4,* and *KLK9* than controls. Other *KLK*s were not significantly expressed.

**Conclusion:**

This study for the first time showed a difference in the expression levels of the *KLK* gene family in recurrent prostate cancer. *KLK*s could be used as recurrence markers for prostate cancer.

Prostate cancer is the most common visceral malignancy and the second deadliest cancer in men. In most cases, the cancer grows gradually, is initially limited to the prostate gland, and does not cause significant morbidity. However, some prostate cancers are aggressive and rapidly spreading (1).

Prostate specific antigen (PSA) or kallikrein (KLK) 3 is an enzyme released into the seminal fluid. It is secreted from the cells surrounding and forming the inner part of the prostate acini and luminal epithelial cells (2). Elevated serum PSA levels are frequently used as prostate cancer markers but are also observed in ejaculation, transurethral catheterization, transrectal ultrasonography, trauma, prostate infections, and benign prostatic hyperplasia. PSA levels can also be used as prognostic markers of cancer recurrence. Such a “biochemical relapse” usually precedes other clinical signs and symptoms of recurrence (3). Biochemical recurrence after radical prostatectomy has been determined using blood PSA level in addition to clinical T stage and Gleason score (4).

*KLK* genes are localized on the 19q13 chromosome and encode 15 serine proteases, the largest protease family in the human genome. These proteases play a role in different physiological processes, such as semen liquefaction and skin shedding. Various amounts of *KLK* genes are expressed in a broad range of tissues, indicating a various degree of their functional involvement in physiological processes (5). For example, certain *KLK* genes (*NES1*, *protease M*, *PSA*) have a reduced expression in the prostate, breast, and some other cancers. *NES1* has been shown to be a potent angiogenesis inhibitor of a new breast cancer tumor suppressor protein and PSA (6).

To the best of our knowledge, no study so far has eveluated *KLK*s expression levels in recurrent and non-recurrent prostate cancer tissues. Therefore, this research aimed to determine the effects of the *KLK* gene family on the recurrence of prostate cancer and evaluate whether KLKs could be used as biomarkers of prostate cancer progression.

## MATERIALS AND METHODS

### Samples

We analyzed 70 recurrent and non-recurrent prostate cancer samples and 19 adjacent healthy prostate tissue samples kindly provided by Baylor College of Medicine, Houston, TX, from 2013 to 2016. The tissues were harvested at radical prostatectomy for clinically diagnosed prostate cancer, snap frozen in liquid nitrogen, and stored at -80 °C as punch biopsies from fresh tissue. The percentage of cancer in the punch area was determined according to published procedures (7). Cancer specimens contained more than 70% tumor tissue, and benign tissue samples were free of cancer or high-grade prostate intraepithelial neoplasia tissue. Recurrence was defined as two successive serum PSA levels above 0.2 ng/mL and was deemed as a biochemical recurrence. All patients were clinically followed-up until PSA recurrence or for a minimum of four years for non-recurrent patients. Patients' clinical and pathological characteristics are summarized in Table 1. Some of these prostate tissues were previously used by our research team to determine the expression profile of cancer stem cell markers, specifically deregulated microRNAs in recurrent and non-recurrent prostate cancer samples (8,9). The study was approved by the Institutional Review Board of Baylor College of Medicine (2018/20-14).

**Table 1:** Clinicopathological characteristics of patients with recurrent and non-recurrent prostate cancer *

	No. of patients		
	with recurrence (N = 34)	without recurrence (N = 36)	*P*
Age, mean ± standard deviation	61.24 ± 1.2	61.42 ± 1.1	0.28
DMOS, mean ± standard deviation	22.6 ± 4.23		<0.001
DMOS mean ± standard deviation		75.25 ± 1.56	
Gleason score			
≤6	4	17	0.004
7 (3 + 4)	14	11	0.216
7 (4 + 3)	10	4	1.000
8-10	6	4	0.453
Race			
African-American	2	1	0.457
Caucasian	29	30	
Hispanic	1	2	
NA	2	3	
Pre-operative PSA	21.73 ± 3.26	9.65 ± 2.84	0.019

*DMOS (R) – months from operation to first recurrence; DMOS (NR) – months from operation to last normal evaluation; NA – not available.

### RNA isolation

For RNA isolation, 1 mL of Trizol solution was added to tissues pulverized in liquid nitrogen. Total RNAs were extracted as per manufacturer’s instructions. RNA purity and concentration were measured spectrophotometrically with optical density measurement using NanoDrop ND-2000c (Thermo Fisher Scientific, Dreieich, Germany) spectrophotometer device with absorbance values of 260 nm and 280 nm wavelengths.

### Complementary DNA synthesis and quantitative RT-PCR

Complementary DNAs (cDNAs) were synthesized with Transcriptor High Fidelity Reverse Transcription kit (Roche, Basel, Switzerland) using the same number of samples from the isolated RNAs. Reverse transcriptase PCR protocol with Oligo (dt) primers for single chain cDNA synthesis was used as per the manufacturer’s protocol. qPCR analysis was performed with SYBR Green Master Mix (Roche, Basel, Switzerland). All primer sequences are shown in Table 2. The mix is optimized for SYBR Green reactions, and the experiments were performed in Light Cycler 480-II (Roche) qRT-PCR. β-actin was used as an internal control. The relative quantification analysis was performed by delta-delta-Ct method as reported previously (10).

**Table 2:** Primer sequences of the kallikrein family

Gene	Sequence
*KLK1-F*	CAGACTTCATGCTGTGTGTCG
*KLK1-R*	TTCTCCGCTATGGTGTCCTC
*KLK2-F*	AGCCTGCCAAGATCACAGAT
*KLK2-R*	CCTTCTCAGAGTAAGCTCTAGCACA
*KLK3-F*	CGTGACGTGGATTGGTGC
*KLK3-R*	GCCGCAGACTGCCCTG
*KLK4-F*	CTCGCTAACGACCTCATGCT
*KLK4-R*	TGCAGACCTCCTCAGACACC
*KLK5-F*	TCCTCTCATTGTCCCTCTGC
*KLK5-R*	CGCAGAACATGGTGTCATCT
*KLK6-F*	GATGGTGGTGCTGAGTCTGA
*KLK6-R*	CCCACAGTGGATGGATAAGG
*KLK7-F*	CTGTCATCCATGGTGAAGAAAGT
*KLK7-R*	TTGACATCCACGCACATGA
*KLK8-F*	GTGGCAACTGGGTCCTTACA
*KLK8-R*	TGCTCTGGGCCATCTTTATT
*KLK9-F*	TCCACCTTACTCGGCTCTTC
*KLK9-R*	GCTGAGGTCCTTGTTGAAGC
*KLK10-F*	TCTCGCTCTTCAACGGCCT
*KLK10-R*	CCCTACTCGAGCCCACAGT
*KLK11-F*	GGCAACATCACAGACACCA
*KLK11-R*	CCCAGGAGATAATGCCTTGA
*KLK12-F*	TGTGTGTTCTTGGGCTCAGC
*KLK12-R*	CCCACCTGTGGTCAATAAGGAC
*KLK13-F*	GCACAAAAGAGGGTGGCAA
*KLK13-R*	CGGATCCACAGGACGTATCT
*KLK14-F*	GCCTATCCTAGAACCATCACG
*KLK14-R*	CTGGAGCTGTCCTCTGCA
*KLK15-F*	GGAAGGTGACGAGTGTGC
*KLK15-R*	TTGCGCAGGTTGTGCTCT
*β-actin-F*	GCCTCGCCTTTGCCGATC
*β-actin-R*	CCCACGATGGAGGGGAAG

### Statistical analysis

Normality testing was performed with the Kolmogorov-Smirnov test. Data are expressed as mean ± standard deviation. Significance of differences in *KLKs* expression between the groups was assessed with the *t* test. Spearman’s rho coefficient was used to assess the correlation between *KLK* genes, serum PSA levels, and Gleason scores. For normally distributed variables, the significance of differences among groups was assessed with one way ANOVA with *post-hoc* Tamhane T2 for the groups in which the variances were not equal. If the variances were homogeneous, the Fisher LSD test was performed. For not normally distributed variables (KLK9), the Kruskal-Wallis H test was used. All significance values were adjusted by the Bonferroni correction. Recurrent and non-recurrent patients were compared according to race with the χ^2^ test. The level of statistical significance was set at *P* ≤ 0.05. Statistical analyses were performed with SPSS v. 16.0 (SPSS Inc., Chicago, IL, USA), and graphs were created with the GraphPad Prism Trial Version (GraphPad, San Diego, CA, USA).

## RESULTS

**Figure 1:**
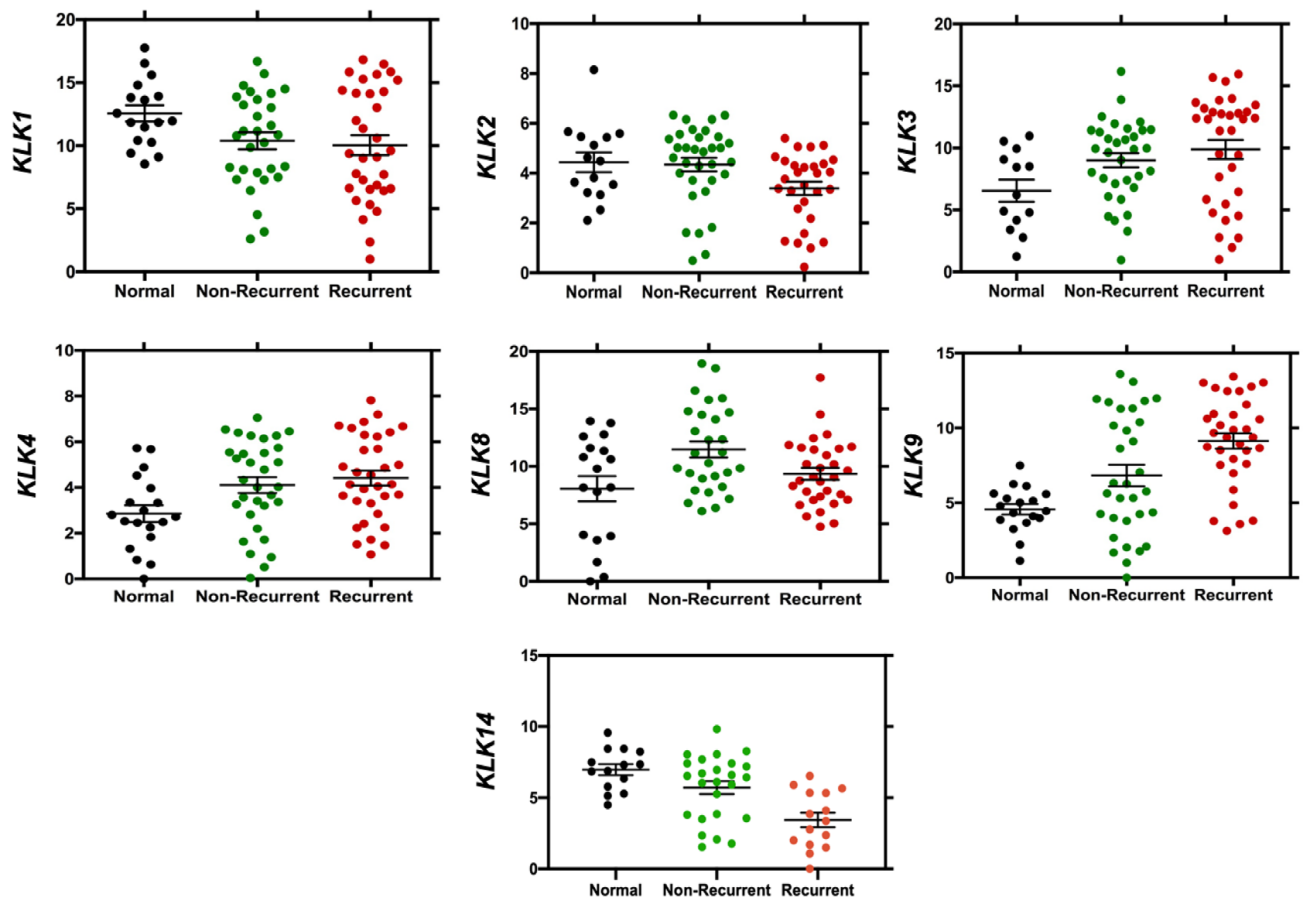
Relative expression levels of *KLK1, KLK2, KLK3, KLK4, KLK8, KLK9,* and *KLK14* in recurrent and non-recurrent tumor tissues.

*KLK1* expression was significantly decreased in recurrent (*P* = 0.048), non-recurrent (*P* = 0.03), and both tumor tissues combined (R+NR) compared with healthy tissues (*P* = 0.007). *KLK2* expression was significantly decreased in recurrent tumor tissues compared with non-recurrent tissues (*P* = 0.021), and in non-recurrent tissues compared with healthy tissues (*P* = 0.026). There was no significant difference between healthy tissues and R + NR tissues. The expression of both *KLK3* and *KLK4* was increased in recurrent tissues (*P* = 0.049, *P* = 0.007, respectively) and R + NR tissues compared with healthy tissues (*P* = 0.025, *P* = 0.005, respectively). *KLK8* expression did not significantly differ between recurrent tissues and healthy tissues and between R + NR tissues and healthy tissues, and it was increased in non-recurrent tissues compared with recurrent tissues (*P* = 0.045). *KLK9* was significantly increased in recurrent tissues (*P* < 0.001) and R + NR tissues compared with healthy tissues (*P* < 0.001). *KLK14* was the only *KLK* significantly decreased in R + NR tissues compared with healthy tissues (*P* = 0.004). *KLK14* was significantly decreased in recurrent tissues compared with healthy tissues (*P* < 0.001) and significantly increased in recurrent tissues compared with non-recurrent tissues (*P* = 0.001) (Figure 1). We did not observe a significant expression differences in *KLK5*, *KLK6*, *KLK7*, *KLK10*, *KLK11*, *KLK12*, *KLK13,* and *KLK15*.

There was no significant correlation between age and Gleason score, except for Gleason score ≤6 and race, whereas pre-operative PSA levels were significantly different between the recurrent and non-recurrent groups (*P* = 0.019, Table 1). There was no correlation between PSA levels and *KLK*s expression, except for *KLK1* and *KLK14,* in the recurrent group. We only found a significant negative correlation between Gleason score and *KLK14* in the non-recurrent group (Table 3).

**Table 3:** Differences in kallikreins expression between tumor and control samples, and correlations between gene expression of kallikreins, serum prostate specific antigen (PSA), and Gleason score*

Relative expression					PSA		Gleason score	
Genes	T/H *P* Value	R/NR *P* Value	R/H *P* Value	R *P* Value		NR *P* Value	R *P* Value (correlation coefficients)	NR *P* Value (correlation coefficients)
***KLK1***	0.007	0.978	0.048	0.026		0.544	0.295 (0.188)	0.103 (-0.303)
***KLK2***	0.906	0.021	0.026	0.842		0.986	0.418 (-0.156)	0.495 (0.123)
***KLK3***	0.025	0.081	0.049	0.665		0.056	0.541 (-0.110)	0.364 (-0.163)
***KLK4***	0.005	0.106	0.007	0.427		0.849	0.666 (-0.780)	0.649 (-0.084)
***KLK5***	0.451	0.249	0.233	0.512		0.246	0.564 (-0.253)	0.526 (0.274)
***KLK6***	0.231	0.315	0.345	0.816		0.267	0.209 (-0.279)	0.895 (0.430)
***KLK7***	0.082	0.245	0.203	0.305		0.283	0.246 (-0.369)	0.269 (0.342)
***KLK8***	0.120	0.045	0.335	0.752		0.842	0.655 (-0.376)	0.093 (0.324)
***KLK9***	<0.001	0.086	<0.001	0.545		0.617	0.745 (-0.584)	0.905 (0.322)
***KLK10***	0.303	0.272	0.023	0.813		0.605	0.767 (-0.465)	0.465 (0.352)
***KLK11***	0.165	0.312	0.392	0.127		0.624	0.115 (-0.478)	0.541 (0.432)
***KLK12***	0.209	0.203	0.065	0.642		0.403	0.234 (-0.336)	0.237 (0.159)
***KLK13***	0.096	0.305	0.245	0.265		0.534	0.426 (-0.257)	0.267 (0.279)
***KLK14***	0.004	0.001	<0.001	0.047		0.586	0.353 (-0.258)	0.049 (-0.398)
***KLK15***	0.712	0.613	0.349	0.635		0.451	0.798 (-0.158)	0.458 (0.536)

*T – tumor; H – healthy; R – recurrent; NR – non-recurrent.

## DISCUSSION

In this study, *KLK1, KLK4, KLK9,* and *KLK14* were strongly decreased in prostate cancer samples compared with controls. Recurrent samples were negative for *KLK1*, *KLK2,* and *KLK14* but demonstrated higher levels of *KLK3, KLK4,* and *KLK9* than controls. Other *KLK*s were not significantly expressed. To the best of our knowledge, this is the first study that evaluated KLKs expression levels in R+NR prostate cancer tissues.

*KLK1* was downregulated in recurrent and non-recurrent prostate cancer tissues compared with healthy samples. This is in contrast to the findings of Mingxin (11), who observed *KLK1* expression in gallbladder cancer, especially in female patients. Other studies reported *KLK2*, *KLK3,* and *KLK4* expression in the breast and prostate (12). The *KLK2* protein product is emerging as a new prostate tumor marker. It activates PSA by converting its pre-form to an active mature form. Lower *KLK2* is also correlated with prostate cancer risk and higher percentage of free PSA, both of which are related to lower total PSA (13). Similar to a previous study, in our study *KLK2* was under-expressed in recurrent prostate cancer tissues compared with healthy tissues (14).

PSA (*KLK3*) plays a vital role in both normal biology and tumor growth and progression (15). In our study, it was overexpressed in recurrent and non-recurrent cancer tissues, as was the case with *KLK4*. Another study (15) also found *KLK4* to be significantly expressed in 55% of ovarian cancer tissue samples. *KLK4* expression was reported to be strongly positively correlated with clinical stage and tumor grade (16).

*KLK5* was reported to be overexpressed in ovarian cancer (17), while in prostate cancer its expression levels were notably decreased compared with control tissues (18). Similar to *KLK5*, *KLK6* is a biomarker for ovarian cancer, but is also overexpressed in colorectal cancers (19). When it comes to *KLK7,* its mRNA was significantly lower in either stage I or stage II breast cancer patients, and its high mRNA expression was related to a favorable prognosis. It was also expressed at unexpectedly elevated levels in ovarian cancer (19). Although in our study *KLK8* was upregulated in both recurrent and non-recurrent prostate cancer tissues, its expression was lower in recurrent than in non-recurrent cancer. Patients with recurrent prostate cancer might have a loss of *KLK8* gene sequences or are affected by other factors. In ovarian tumors, the expression of the *KLK8* and its spliced variants indicate the frequent expression of novel variants. This full-length *KLK8* expression is an independent and favorable marker for ovarian cancer (20).

*KLK9* in our study was upregulated in recurrent and non-recurrent prostate cancer tissues. Other studies observed elevated *KLK9* expression in the early stages of breast cancer compared with advanced stages and in patients with tumor size <2 cm compared with bigger tumors (21). In addition, higher *KLK9* expression is a favorable prognostic marker of ovarian cancer (22). Elevated levels of *KLK11* are found in the prostate, gastric tissue, trachea, colon, and skin samples (23). When it comes to *KLK13*, a recently identified family member of kallikreins, it is significantly upregulated in metastatic lung adenocarcinoma. *KLK13* overexpression is reported to result in an increased tumor malignant potential, knockdown of its internal gene expression, and decreased cell migration and invasive characteristics. Functional studies further showed that *KLK13* was activated via de-methylation of its upstream site (24).

Another study showed that seven genes (*KLK5*-*8*, *KLK10*, *KLK11*, and *KLK14*) were elevated in ovarian cancer tissue samples and cell lines compared with the healthy ovary (25). *KLK12*, *KLK13,* and *KLK14* genes were downregulated in breast cancer (17), while *KLK15* was overexpressed in more aggressive forms of prostate cancer (26). Another study has shown trypsin-like activity of *KLK15* in prostate cell line (27), but we did not observe a significant change related to this gene.

Our results point to the fact that *KLK1*, *KLK2*, and *KLK14* (only in recurrent prostate cancer tissues) are underexpressed in prostate cancer tissues, while *KLK3*, *KLK4*, *KLK8*, and *KLK9* are overexpressed in both recurrent and non-recurrent tissues. The limitations of this study include a relatively small size, retrospective study design, and a single-center experience.

In conclusion, we propose that *KLK1, KLK2, KLK3 KLK4, KLK8, KLK9*, and *KLK14,* which are differentially expressed in prostate cancer, could be used as promising biomarkers of prostate cancer progression. In our opinion, KLKs play a role in promoting or inhibiting tumor growth and metastasis by regulated gene expression. However, our findings need to be validated in studies with a much larger number of participants. In addition, more novel markers should be assessed.
